# Levocarnitine Improves AlCl_3_-Induced Spatial Working Memory Impairment in *Swiss albino* Mice

**DOI:** 10.3389/fnins.2019.00278

**Published:** 2019-03-26

**Authors:** Md. Mamun Al-Amin, Md. Irfan Amin Chowdury, A. R. M. Saifullah, Mohammed Nazmul Alam, Preeti Jain, Murad Hossain, Md. Ashraful Alam, Mohsin Kazi, Ajaz Ahmad, Mohammad Raish, Abdulmohsen Alqahtani, Hasan Mahmud Reza

**Affiliations:** ^1^Department of Pharmaceutical Sciences, North South University, Dhaka, Bangladesh; ^2^Department of Pharmaceutics, College of Pharmacy, King Saud University, Riyadh, Saudi Arabia; ^3^Department of Clinical Pharmacy, College of Pharmacy, King Saud University, Riyadh, Saudi Arabia

**Keywords:** levocarnitine, Alzheimer’s disease, working memory, neurotoxicity, oxidative stress markers, antioxidants

## Abstract

**Background:** Aluminum, a neurotoxic substance, causes oxidative stress induced-neurodegenerative diseases. Several lines of evidence suggest that levocarnitine has an antioxidant effect and also plays an important role in beta-oxidation of fatty acids. However, the role of levocarnitine in aluminum-induced neurotoxicity has not been well documented. Here we aimed to investigate the effect of levocarnitine on aluminum chloride (AlCl_3_)-induced oxidative stress and memory dysfunction.

**Methods:** Male *Swiss albino* mice (*n* = 30) were treated with either control (saline) or AlCl_3_ or AlCl_3_ plus levocarnitine or levocarnitine or astaxanthin plus AlCl_3_ or astaxanthin alone. The spatial working memory was determined by radial arm maze (RAM). In addition, we measured the lipid peroxidation (MDA), glutathione (GSH), advanced oxidation of protein products (AOPP), nitric oxide (NO) and activity of superoxide dismutase (SOD) in the various brain regions including prefrontal cortex (PFC), striatum (ST), parietal cortex (PC), hippocampus (HIP) hypothalamus (HT) and cerebellum (CB). We used astaxanthin as a standard antioxidant to compare the antioxidant activity of levocarnitine.

**Results:** The RAM data showed that AlCl_3_ treatment (50 mg/kg) for 2 weeks resulted in a significant deficit in spatial learning in mice. Moreover, aluminum exposure significantly (*p* < 0.05) increased the level of oxidative stress markers such as MDA, GSH, AOPP and NO in the various brain regions compared to the controls. In addition, combined administration of levocarnitine and AlCl_3_ significantly (*p* < 0.05) lowered the MDA, AOPP, GSH and NO levels in mice.

**Conclusion:** Our results demonstrate that levocarnitine could serve as a potential therapeutic agent in the treatment of oxidative stress associated diseases as well as in memory impairment.

## Introduction

Homeostasis of metal ions is highly important for normal cognitive function. The homeostatic dysregulation has been considered as the key factor in the progression of neurodegenerative diseases (Nelson, 1999). Aluminum is widely used as a household product, and excessive use of aluminum has been found to be associated with bone, blood and brain disorders (Han et al., 2013). Earlier investigations revealed aluminum as a major risk factor in the development of amyotrophic lateral sclerosis, dementia, Parkinsonism and Alzheimer’s diseases (Kawahara, 2005; Singla and Dhawan, 2014). Aluminum exposure causes alternation of monoamine imbalance especially in cholinergic and noradrenergic neurotransmission and also leads to the generation of free radical species through disruption of glucose metabolism (LeBel and Bondy, 1991; Kawahara, 2005).

Aluminum has been shown to cause oxidative stress (OS)-induced neurodegeneration through iron accumulation and reactive oxygen species (ROS) formation (Wu et al., 2012). ROS alters the level of antioxidant enzymes such as catalase (CAT) and superoxide dismutase (SOD), and it was reported that the activity of SOD decreased in the hippocampus and cerebral cortex of the brain in response to the oxidative stress (Sitta et al., 2009). Different doses of aluminum lead to region-specific oxidative DNA damage in rats (Bodea et al., 2010) and mice (Augustyniak and Skrzydlewska, 2009). Aluminum exposure has also been reported to increase lipid peroxidation (Lee et al., 2014; Al-Amin et al., 2016) and decrease the level of glutathione (Dutta et al., 2008).

Several compounds with antioxidant properties are used for the treatment of oxidative stress- induced neurodegenerative disorders including cognitive dysfunction. Levocarnitine (4-N-trimethylammonium-3-hydroxybutyric acid) is a natural nutrient which prevents toxic accumulation of long chain fatty acids (LCFA) by transporting them into mitochondria (Sinclair et al., 2005). Levocarnitine supplementation had shown antioxidant effects in patients with coronary artery disease by improving malondialdehyde (MDA), CAT, SOD and glutathione (GSH) levels (Lee et al., 2014). It has been reported earlier that levocarnitine decreases oxidative stress by improving metabolic function (Hagen et al., 2002). We hypothesized that levocarnitine would exhibit the antioxidant effect in the brain tissues as well. In this study, we aimed to combinedly administer levocarnitine and aluminum chloride to understand whether levocarnitine could prevent aluminum chloride induced spatial working memory deficits and oxidative stress in mice. The experimental results were compared with a standard antioxidant drug, astaxanthin which is more potent than conventional antioxidants α-tocopherol and β-carotene (Miki, 1991). In addition, we previously showed that astaxanthin improved aluminum chloride induced oxidative stress in several brain regions including, prefrontal cortex, striatum, hypothalamus, hippocampus and cerebellum (Al-Amin et al., 2016).

## Materials and Methods

### Experimental Animals

*Swiss albino* mice (30–35 g and 8 weeks old) were used in this study. They were housed at 25°C under adequate light condition with standard mice pellets and water. Mice were randomly divided into four groups. Control groups were treated with saline while test groups were treated with levocarnitine (LC) (50 mg/kg) for 6 weeks. All the experimental procedures were approved by the local ethical committee of the North South University for animal experiments (*Ref*: ERC-IACUC/NSU/026-15).

### Experimental Design

The drugs were administered orally once daily for 6 weeks. Animals were divided into the following six groups:

(1) Control (*n* = 6): Control groups received 1,000 μl of 0.9% saline.
(2) AlCl_3_ (*n* = 6): Aluminum chloride was given orally at a dose of 50 mg/kg body weight per day (Al-Amin et al., 2016). Aluminum chloride was dissolved in distilled water (50 mg/20 ml). The mice were given 1,200- 1,500 μl of aluminum chloride solution depending on body weight.
(3) LC (*n* = 6): Levocarnitine at a dose of 50 mg/kg body weight was given (Akisu et al., 2002). Levocarnitine was dissolved in distilled water (50 mg/20 ml). The mice were administered 1,200, –1,500 μl of levocarnitine solution depending on body weight.
(4) AlCl_3_-LC (*n* = 6): Levocarnitine was given orally at a dose of 50 mg/kg body weight (Akisu et al., 2002) together with aluminum chloride at a dose 50 mg/kg body weight (Singh and Goel, 2015).
(5) AST (*n* = 3): Astaxanthin at a dose of 20 mg/kg body weight was given (Pei et al., 2008; Al-Amin et al., 2016). Astaxanthin powder was dissolved in distilled water (20 mg/20 ml). The mice were given 500–600 μl of astaxanthin solution depending on body weight.
(6) AlCl_3_-AST (*n* = 3): Astaxanthin was given orally at a dose of 20 mg/kg body weight (Pei et al., 2008; Al-Amin et al., 2016) together with aluminum chloride at a dose 50 mg/kg body weight (Singh and Goel, 2015).

After 6 weeks of treatment, we measured spatial working memory using the 8-arm radial arm maze. The animals were then euthanized and brain tissues were collected to measure a range of oxidative stress markers.

### Radial Arm Maze Test

Eight-arm radial maze was constructed following the measurements as described by Terragon et al. (2012).

#### Training

We used food pellets as a target to train the animals. The task of the mice was to find the target making fewer mistakes during the training session. Each mouse was placed in the center of the RAM device to find out the target for 10 min. Initially, all the arms contained the target. The target was placed at the end of the arm within a well. However, the target-containing arms were reduced gradually. The training sessions consisted of 8 consecutive days; Day 1,2: all arms contain target; Day 3: 4 arms; Day 4,5: 3 arms; Day 6: 2 arms; Day 7,8: 1 arm. The training was stopped once the animals visited all of the arms or spent 10 min. The successful performers (mice) were finally included for the experiments.

#### Testing Session

On the day of the experiment, only one arm contained food and all the animals were kept on overnight fasting (except water). Mice were released at the center of the maze and allowed to explore the maze. Parameters were followed as stated by Terragon et al. (2012) and three trials were run for each mouse. Each trial was recorded using the webcam for manual analysis later. After each run, the apparatus was cleaned with 70% ethanol. The spatial working memory data analysis was performed based on our previously established protocol (Al-Amin et al., 2014, 2016).

### Tissue Processing

Mice were anesthetized using 200 μl of ketamine (50 mg/ml, ACI pharmaceuticals Ltd., Bangladesh). The animals were sacrificed by decapitation. The brain was immediately removed from the skull and immediately transferred to a petri dish placed over ice. The hippocampal, striatum, prefrontal cortex, parietal cortex, hypothalamus and cerebellum tissues were microdissected and preserved in –20°C.

The homogenates of brain tissues 10% (w/v) were prepared in sodium phosphate buffer (1× PBS pH 7.0) using Ultra-Turrax T25 (United States) homogenizer. Homogenized tissue samples were sonicated at 5-s cycle for 150 s using an ultrasonic processor. The homogenized samples were centrifuged at 10,000 rpm (7960 *g*) for 10 min at 4°C. The clear supernatants were collected and stored at –20°C for the biochemical analysis.

### Oxidative Stress Measurement

#### Estimation of Lipid Peroxidation (MDA)

Lipid peroxidation was estimated colorimetrically by measuring thiobarbituric acid reactive substances (TBARS) as described previously (Niehaus and Samuelsson, 1968). Briefly, 0.1 ml of tissue homogenate in Tris–HCl buffer (pH 7.5) was treated with 2 ml of TBA-TCA-HCl (1:1:1 ratio) reagent (thiobarbituric acid 0.37%, 0.25 N HCl and 15% TCA) and placed in water bath for 15 min and cooled. The absorbance of the clear supernatant was measured against reference blank at 535 nm (Al-Amin et al., 2014). The level of MDA was measured by using standard curve and expressed as nmol/ml.

#### Advanced Oxidation of Protein Products (AOPP)

Advanced oxidation of protein products were determined spectrophotometrically (Witko-Sarsat et al., 1996; Al-Amin et al., 2015a). Briefly, 50 μl of plasma diluted with phosphate-buffered saline (PBS) at a ratio of 1:2 and chloramine T (0–100 mmol/L) were used for the preparation of the calibration curve. PBS was used as a blank. 100 μl of 1.16 M potassium iodide and 50 μl of acetic acid were added to each well and absorbance at 340 nm was measured immediately. Concentrations of APOPs were expressed in chloramine units (μmol/ml).

#### Glutathione (GSH) Level

Glutathione in the brain was assayed according to a previously described method (Ellman, 1959; Al-Amin et al., 2015b). Briefly, 2.7 ml of phosphate buffer (0.1 M, pH 8) and 0.2 ml of 5, 5-dithio-bis (2-nitrobenzoic acid) were added with 1 ml of plasma. The color developed was measured immediately at 412 nm. Results were expressed as μmol/mg protein.

#### Nitric Oxide (NO) Level

The levels of Nitric Oxide (NO) was measured based on a previous protocol (Tracey et al., 1995) using the Griess-Illosvoy reagent. The tissue homogenates were diluted with PBS (2:8 ratio) and incubated at 25°C for 15 min in a 96-well plate (Al-Amin et al., 2015b). The absorbances were then measured at a wavelength of 540 nm against the blank readings of the spectrophotometer.

#### Assay of Superoxide Dismutase (SOD) Activity

The activity of SOD was assayed by a modified procedure as described previously (Ma et al., 2010; Al-Amin et al., 2018a). Briefly, the reaction mixture contained 50 mM sodium phosphate (pH 7.8), 13 mM methionine, 75 mM nitroblue tetrazolium (NBT), 2 mM riboflavin, 100 mM EDTA and 2 mL of plasma. The change in absorbance of each sample was then recorded at 560 nm after the formation of the blue formazan.

### Statistical Analysis

One-way ANOVA was conducted to measure the main effect of the treatment groups. *Post hoc* test namely, “Newman-Keuls” was used to compare the difference between groups. All analyses were carried out with the Graph pad prism (version 6.0) software. The difference was considered significant when the *p*-value was found less than 0.05. Data are represented as mean ± SEM (Standard Error of the Mean).

## Results

### Effect of Various Treatments on Working Memory Performance in Radial Arm Maze Test

**Figure 1:**
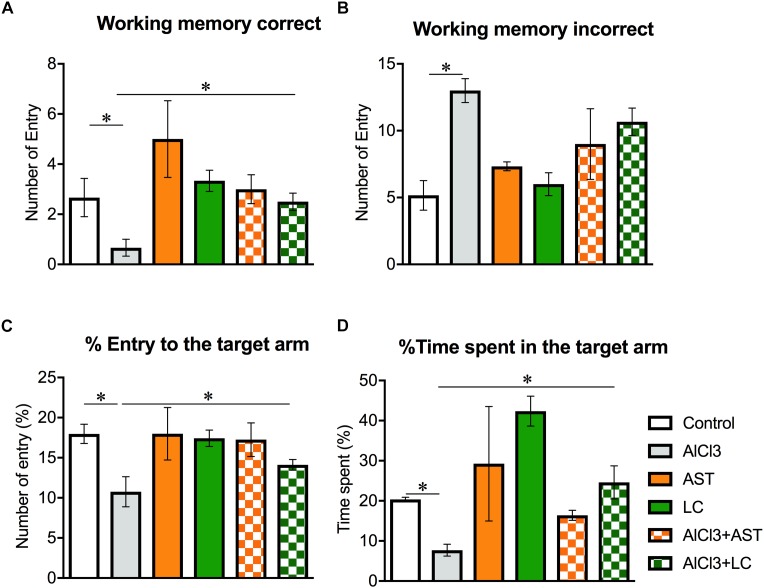
Effect of levocarnitine and aluminum chloride on spatial working memory. The aluminum treated mice showed a lower working memory **(A)**; a higher incorrect working memory **(B)**; a lower percentage of entry to the target arm **(C)**; and a higher number of arm entry **(D)**. The combined (levocarnitine plus aluminum) treatment improved these parameters. The groups were control, AlCl3 (aluminum chloride) and AlCl3+LC (aluminum chloride and levocarnitine) and LC (levocarnitine). Data presented as mean ± SEM. *n* = 6 per group except AST (*n* = 3); and AlCL3+AST (*n* = 3); ^∗∗^*p* < 0.01, ^∗^*p* < 0.05.

There was a significant main effect of treatment on the working memory correct [*F*_(5,24)_ = 4.58; *p* = 0.004] (Figure 1A) and working memory incorrect [*F*_(5,24)_ = 7.90; *p* < 0.001] (Figure 1B). However, student *t*-test showed that the number of “working memory correct” was significantly (*p* < 0.05) decreased in the AlCl_3_-treated mice compared to control mice. In addition, the number of “working memory incorrect” was also significantly (*p* < 0.05) increased in AlCl_3_ treated mice compared to the control mice. Surprisingly, AlCl_3_+LC-treated mice showed increased entry to the target arm than AlCl_3_-treated mice, indicating an improvement of working memory. We also observed a significant main effect of treatment on the “percentage entry to the target arm” [*F*_(5,24)_ = 3.93; *p* < 0.001] (Figure 1C) and “time spent in the target arm” [*F*_(5,24)_ = 8.50 *p* < 0.001] (Figure 1D). The results of this RAM test suggest that levocarnitine intake may facilitate the finding of the target, suggesting an improvement of the spatial working memory performance. The result has been compared with the standard drug AST.

### Biochemical Estimation

#### Lipid Peroxidation (MDA Levels)

**Figure 2:**
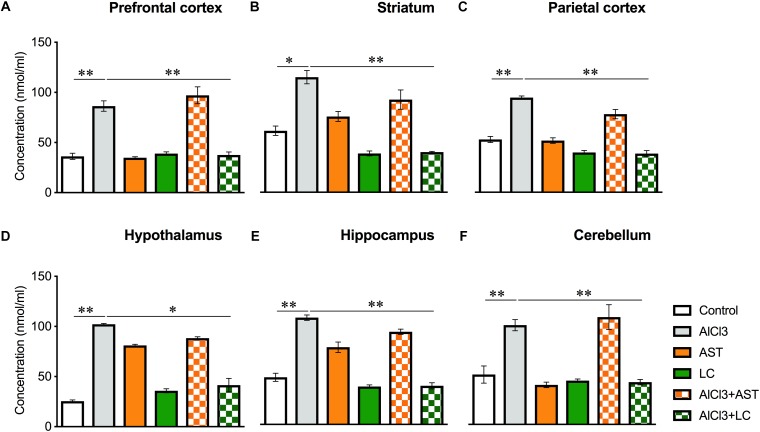
Treatment of levocarnitine and aluminum chloride on MDA (lipid peroxidation) **(A–F)**; in the prefrontal cortex (PFC) **(A)**, striatum (ST) **(B)**, parietal cortex (PC) **(C)**, hypothalamus (HT) **(D)**, hippocampus (HP) **(E)** and cerebellum (CB) **(F)**. The groups were control, AlCl3 (aluminum chloride), AST (astaxanthin), AST+AlCl3 (aluminum chloride and astaxanthin) and AlCl3+LC (aluminum chloride and levocarnitine) and LC (levocarnitine). Data presented as mean ± SEM. *n* = 6 per group except AST (*n* = 3); and AlCL3+AST (*n* = 3); ^∗∗∗^*p* < 0.001, ^∗∗^*p* < 0.01, ^∗^*p* < 0.05.

Our results showed a significant main effect of treatments on the MDA levels in the prefrontal cortex (PFC) [*F*_(5,16)_ = 49.58, *p* < 0.001] (Figure 2A); striatum (ST) [*F*_(5,21)_ = 33.82, *p* < 0.001] (Figure 2B); parietal cortex (PC) [*F*_(5,21)_ = 76.39, *p* < 0.001] (Figure 2C); hypothalamus (HT) [*F*_(5,22)_ = 240.0, *p* < 0.001] (Figure 2D); hippocampus (HIP) [*F*_(5,22)_ = 90.0, *p* < 0.001] (Figure 2E) and cerebellum (CB) [*F*_(5,17)_ = 19.30, *p* < 0.001] (Figure 2F). The *post hoc* test indicated that the level of MDA was noticeably (*p* < 0.05) increased in the aluminum-exposed group in the PFC, ST, PC, HT, HIP and CB. On the other hand, levocarnitine treatment in aluminum exposed group improved the levels of MDA in these brain regions. AST has been used as reference standard.

#### Advanced Oxidation of Protein Products (AOPP)

**Figure 3:**
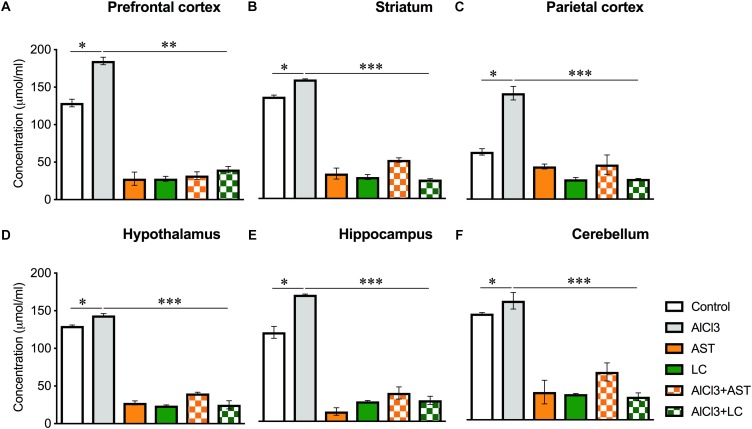
Treatment of levocarnitine and aluminum chloride on advanced oxidation of protein produces (AOPP) **(A–F)**; in the prefrontal cortex (PFC) **(A)**, striatum (ST) **(B)**, parietal cortex (PC) **(C)**, hypothalamus (HT) **(D)**, hippocampus (HP) **(E)** and cerebellum (CB) **(F)**. The groups were control, AlCl3 (aluminum chloride), AST (astaxanthin), AST+AlCl3 (aluminum chloride and astaxanthin) and AlCl3+LC (aluminum chloride and levocarnitine) and LC (levocarnitine). Data presented as mean ± SEM. *n* = 6 per group except AST (*n* = 3); and AlCL3+AST (*n* = 3); ^∗∗∗^*p* < 0.001, ^∗∗^*p* < 0.01, ^∗^*p* < 0.05.

Our results showed a significant main effect of treatment on the AOPP levels in the PFC [*F*_(5,22)_ = 177.2, *p* < 0.001] (Figure 3A); ST [*F*_(5,21)_ = 412.2, *p* < 0.001] (Figure 3B); PC [*F*_(5,21)_ = 50.26, *p* < 0.001] (Figure 3C); HT [*F*_(5,21)_ = 643, *p* < 0.001] (Figure 3D); HIP [*F*_(5,21)_ = 138.4, *p* < 0.001] (Figure 3E) and in the CB [*F*_(5,)_ = 138.4, *p* < 0.001] (Figure 3F). The *post hoc* test indicated that the level of AOPP was markedly (*p* < 0.05) elevated in the PFC, ST, PC, HT, HIP of the aluminum-exposed group except for CB. Interestingly, exposure with aluminum plus levocarnitine improved the levels of AOPP in these brain regions. The standard drug AST has been used to compare the result.

#### Glutathione (GSH)

**Figure 4:**
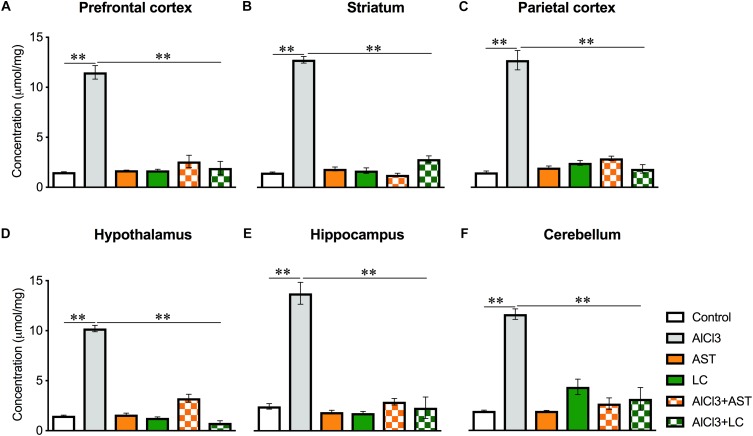
Treatment of levocarnitine and aluminum chloride on glutathione (GSH) **(A–F)**; in the prefrontal cortex (PFC) **(A)**, striatum (ST) **(B)**, parietal cortex (PC) **(C)**, hypothalamus (HT) **(D)**, hippocampus (HP) **(E)** and cerebellum (CB) **(F)**. The groups were control, AlCl3 (aluminum chloride), AST (astaxanthin), AST+AlCl3 (aluminum chloride and astaxanthin) and AlCl3+LC (aluminum chloride and levocarnitine) and LC (levocarnitine). Data presented as mean ± SEM. *n* = 6 per group except AST (*n* = 3); and AlCL3+AST (*n* = 3); ^∗∗∗^*p* < 0.001, ^∗∗^*p* < 0.01, ^∗^*p* < 0.05.

A significant main effect of various treatments was observed on PFC [*F*_(5,21)_ = 102.9, *p* < 0.001] (Figure 4A); ST [*F*_(5,22)_ = 340.7, *p* < 0.001] (Figure 4B); PC [*F*_(5,21)_ = 104.5, *p* < 0.001] (Figure 4C); HT [*F*_(5,22)_ = 278.2, *p* < 0.001] (Figure 4D); HIP [*F*_(5,21)_ = 73.41, *p* < 0.001] (Figure 4E) and CB [*F*_(5,20)_ = 28.74, *p* < 0.01] (Figure 4F). The *post hoc* test indicated that the levels of GSH in the PFC, ST, PC, HT, HIP and CB were markedly (*p* < 0.05) higher in the aluminum-exposed group than the control group. However, exposure with aluminum plus levocarnitine reversed the levels of GSH in these brain regions. The result has been compared with the standard drug AST.

#### Nitric Oxide (NO)

**Figure 5:**
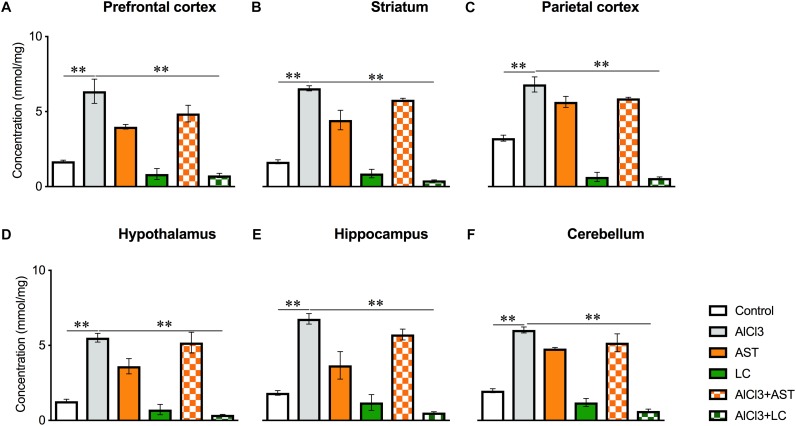
Treatment of levocarnitine and aluminum chloride on nitric oxide (NO) **(A–F)**; in the prefrontal cortex (PFC) **(A)**, striatum (ST) **(B)**, parietal cortex (PC) **(C)**, hypothalamus (HT) **(D)**, hippocampus (HP) **(E)** and cerebellum (CB) **(F)**. The groups were control, AlCl3 (aluminum chloride), AST (astaxanthin), AST+AlCl3 (aluminum chloride and astaxanthin) and AlCl3+LC (aluminum chloride and levocarnitine) and LC (levocarnitine). Data presented as mean ± SEM. *n* = 6 per group except AST (*n* = 3); and AlCL3+AST (*n* = 3); ^∗∗∗^*p* < 0.001, ^∗∗^*p* < 0.01, ^∗^*p* < 0.05.

Our results showed a significant main effect of treatments on the NO levels in the PFC [*F*_(5,24)_ = 25.2, *p* < 0.001] (Figure 5A); ST [*F*_(5,24)_ = 99.65, *p* < 0.001] (Figure 5B); PC [*F*_(5,24)_ = 71.80, *p* < 0.001] (Figure 5C); HT [*F*_(5,24)_ = 43.86, *p* < 0.001] (Figure 5D); HIP [*F*_(5,24)_ = 40.51, *p* < 0.001] (Figure 5E) and in the CB [*F*_(5,24)_ = 115.4, *p* < 0.001] (Figure 5F). The *post hoc* test indicated that the level of NO was markedly (*p* < 0.05) raised in the PFC, ST, PC, HT, HIP and CB of the aluminum-exposed group. On the other hand, exposure with aluminum plus levocarnitine improved the levels of NO in these brain regions. The result has been compared with the standard drug AST.

#### Superoxide Dismutase (SOD)

**Figure 6:**
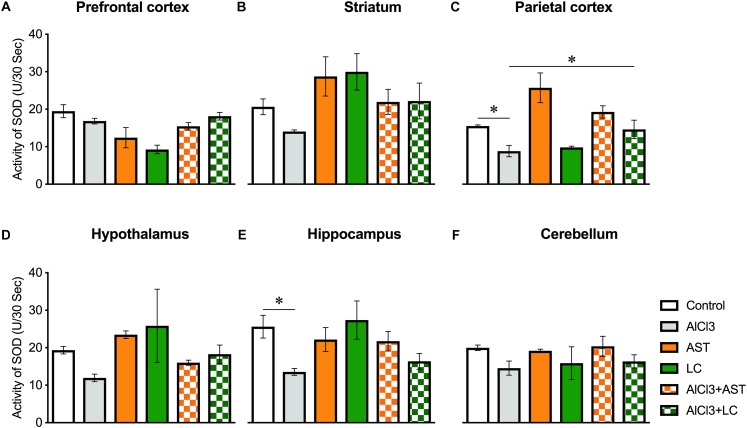
Treatment of levocarnitine and aluminum chloride on superoxide dismutase (SOD) **(A–F)**; in the prefrontal cortex (PFC) **(A)**, striatum (ST) **(B)**, parietal cortex (PC) **(C)**, hypothalamus (HT) **(D)**, hippocampus (HP) **(E)** and cerebellum (CB) **(F)**. The groups were control, AlCl3 (aluminum chloride), AST (astaxanthin), AST+AlCl3 (aluminum chloride and astaxanthin) and AlCl3+LC (aluminum chloride and levocarnitine) and LC (levocarnitine). Data presented as mean ± SEM. *n* = 6 per group except AST (*n* = 3); and AlCL3+AST (*n* = 3); ^∗∗∗^*p* < 0.001, ^∗∗^*p* < 0.01, ^∗^*p* < 0.05.

Results of one-way ANOVA analysis showed a significant main effect of treatments on PFC [*F*_(5,22)_ = 8.14, *p* < 0.001] (Figure 6A); PC [*F*_(5,21)_ = 8.41, *p* < 0.01)] (Figure 6C) and HIP [*F*_(5,24)_ = 3.06, *p* < 0.02] (Figure 6E); but not ST [*F*_(5,24)_ = 2.45, *p* = 0.06] (Figure 6B); HT [*F*_(5,19)_ = 1.73, *p* = 0.17] (Figure 6D); and CB [*F*_(5,24)_ = 0.83, *p* = 0.53] (Figure 6F). The *post hoc* test indicated that the level of SOD in the PC was markedly (*p* < 0.05) lowered in the aluminum-exposed group as compared to the control group. Interestingly, exposure to aluminum plus levocarnitine reversed the levels of SOD in PC. AST has been used as reference standard.

## Discussion

We aimed to investigate the impact of levocarnitine on memory performance and oxidative stress in the PFC, ST, PC, HT, HIP and CB of the AlCl_3_-treated mice. Our study revealed several important findings. First, we found that aluminum exposure leads to impairment of spatial working memory in mice. Second, the concurrent administration of levocarnitine and aluminum improves the spatial working memory performance, suggesting that levocarnitine may have a role in preventing aluminum-induced behavioral impairment. Third, the results of our oxidative stress associated study further support the behavioral data obtained, indicating that the spatial working memory deficit could be associated with the oxidative stress in the cortical and subcortical brain tissues. Fourth, the effect of LC on spatial working memory and oxidative stress was comparable to AST, a potent antioxidant, when concurrently administered with aluminum chloride (Table 1).

**Table 1:** Summary of the *p* values showing comparative effects of treatments between the groups of interest.

Parameter	Control vs. AlCl3	AlCl3 vs. AlCl3+AST	AlCl3 vs. AlCl3+LC	AlCl3+LC vs. AlCl3+AST
**Radial arm maze test**				
Working memory correct	^∗^	^∗^	^∗^	–
Working memory incorrect	^∗∗∗^	^∗^	–	–
% Entry to the target arm	–	–	–	–
% Time spent in the target arm	–	–	^∗^	–
**Oxidative stress**				
**MDA**				
Prefrontal cortex	^∗∗∗^	^∗∗∗^	–	↓^∗^
Striatum	^∗∗∗^	^∗∗∗^	^∗^	↓
Parietal Cortex	^∗∗∗^	^∗∗∗^	^∗∗∗^	↓
Hypothalamus	^∗∗∗^	^∗∗∗^	^∗∗∗^	↓
Hippocampus	^∗∗∗^	^∗∗∗^	^∗∗^	↓
Cerebellum	^∗∗∗^	^∗∗∗^	–	↓
**AOPP**				
Prefrontal cortex	^∗∗∗^	^∗∗∗^	^∗∗∗^	–
Striatum	^∗∗∗^	^∗∗∗^	^∗∗∗^	↓
Parietal Cortex	^∗∗∗^	^∗∗∗^	^∗∗∗^	–
Hypothalamus	^∗∗^	^∗∗^	^∗∗∗^	^∗^
Hippocampus	^∗∗∗^	^∗∗∗^	^∗∗∗^	–
Cerebellum	–	^∗∗∗^	^∗∗∗^	–
**GSH**				
Prefrontal cortex	^∗∗∗^	^∗∗∗^	^∗∗∗^	–
Striatum	^∗∗∗^	^∗∗^	^∗∗∗^	↑
Parietal Cortex	^∗∗∗^	^∗∗∗^	^∗∗∗^	–
Hypothalamus	^∗∗∗^	^∗∗∗^	^∗∗∗^	↓
Hippocampus	^∗∗∗^	^∗∗∗^	^∗∗∗^	–
Cerebellum	^∗∗∗^	^∗∗∗^	^∗∗∗^	–
**NO**				
Prefrontal cortex	^∗∗∗^	^∗^	^∗∗∗^	↓
Striatum	^∗∗∗^	–	^∗∗∗^	↓^∗^
Parietal Cortex	^∗∗∗^	–	^∗∗∗^	↓^∗^
Hypothalamus	^∗∗∗^	–	^∗∗∗^	↓^∗^
Hippocampus	^∗∗∗^	–	^∗∗∗^	↓^∗^
Cerebellum	^∗∗∗^	–	^∗∗∗^	↓^∗^
**SOD**				
Prefrontal cortex	–	–	–	–
Striatum	–	–	–	–
Parietal Cortex	^∗^	^∗∗^	^∗^	–
Hypothalamus	–	–	–	–
Hippocampus	–	–	–	–
Cerebellum	–	–	–	–

*↓ Decrease; ↑ Increase; ^∗^*p* < 0.05; ^∗∗^*p* < 0.01; ^∗∗∗^*p* < 0.001*.

Radial arm maze test is purely a hippocampal-dependent test to measure the hippocampal-dependent spatial working memory (Zhang and O’Donnell, 2000; Sun et al., 2007; Hodges et al., 2009; Al-Amin et al., 2016). In this experiment, the task of the mice was to find out the target making fewer errors (incorrect entry). The fastest re-entry to the target arm indicates that mice encode the spatial information of the baited arm (arm containing pellet) as the target was not visible with the direct eyesight, and the animals had to depend on the hippocampal encoding and retrieval of spatial memory. In radial arm maze test, our results showed that aluminum-exposed animals made more mistakes in finding the target. On the other hand, aluminum-exposed animals visited arms that contained no target. This result suggests that aluminum treatment causes impaired spatial working memory formation. Administration of aluminum chloride also resulted in deterioration of the spatial memory as determined by the radial arm maze test. Our results are in line with a previous report that showed oral administration of aluminum impaired the spatial working memory formation in *Swiss albino* mice (Al-Amin et al., 2016). Also, an intracerebral administration of aluminum chloride caused the spatial learning deficit in the Morris water maze task in rabbits (Goo et al., 2012).

To be noted that in this study all animals were previously trained before administration of drugs. However, levocarnitine plus aluminum-treated mice remembered food containing arms efficiently. Radial-arm maze (RAM) test is a sensitive and reliable way to assess both learning and memory in a variety of species such as rats and mice (Levin, 2015). RAM test device has seven options to make a mistake to enter into the wrong arm. We observed that levocarnitine plus aluminum-treated animals picked up the food spending less time as compared to the aluminum group and also, levocarnitine plus aluminum-treated animals spent more time in the food containing arms. Our findings are also consistent with a previous study demonstrating the learning memory performance (Ritter and Cummings, 2015). Taken together, it is possible that aluminum could cause spatial working memory deficit in animals, while levocarnitine prevents the aluminum-induced decrement of spatial working memory, suggesting that this drug may have a beneficial effect on spatial cognition. However, we do not know the exact mechanism of the spatial working memory impairment caused by aluminum exposure. One possibility may be an increment of oxidative stress (Al-Amin et al., 2016). Since free radicals mediate oxidative damage, it is necessary to investigate the endogenous antioxidant enzymes like superoxide dismutase and glutathione which are the first line defense against free radical damage under oxidative stress.

When the impact of LC was compared with AST on aluminum-induced spatial working memory deficit, we did not find any significant difference. Previously, we reported that AST improves aluminum chloride- (Al-Amin et al., 2016) and scopolamine-induced (Al-Amin et al., 2018b) spatial working memory deficits in *Swiss albino* mice in RAM task. It is possible that the underlying basis of this behavioral finding is similar.

In our study, administration of aluminum chloride resulted in marked oxidation stress as indicated by an increase in lipid peroxidation and nitrite concentrations, and a decrease in glutathione and superoxide dismutase. These changes could have been due to the reduced axonal mitochondrial turnover and disruption of the Golgi induced by aluminum treatment, which resulted in the release of oxidative products like malondialdehyde, carbonyls and peroxynitrites within the neurons. Under oxidative stress, MDA is considered one of the key intermediates of free radical-induced damage. Increased level of MDA has been reported in the cortex, hippocampus, cerebellum in the neurons and astrocyte of aged rodents. MDA interferes with the brain homeostasis between an inhibitory and excitatory neuron (Vaishnav et al., 2010); impairs the function of the brain mitochondria (Li et al., 2012) which results in the disturbances of brain function. Our study showed a significantly increased MDA level in aluminum chloride-treated animals while lower MDA level in all the tested regions of the brain of the levocarnitine-treated mice. These results are consistent with the previous report showing similar findings (Harooni et al., 2009). In addition, levocarnitine treatment was found to reduce MDA level in humans (Atalay Guzel et al., 2015).

Advanced Oxidation of Protein Product is also used as a marker of oxidative stress which is formed when ROS oxidizes the protein products such as oxidized albumin. In this study aluminum chloride exposure significantly increased the level of AOPP, while treatment of levocarnitine strongly reversed this effect. This drastic lowering effect by levocarnitine may be due to its potent antioxidant property that scavenges the level of free radicals produced in the brain tissues. The current results are consistent with the previous report by Orihuela et al. (2005). Glutathione is the most abundant intracellular antioxidant found in reduced form and is involved in direct scavenging of free radicals or serving as a substrate for the glutathione peroxidase enzyme that catalyzes the detoxification of hydrogen peroxide. Our findings that aluminum chloride exposure significantly increased the level of GSH, while treatment of levocarnitine reversed this action are again consistent with a previous report (Kumar et al., 2011). Superoxide dismutase presents the first line of defense against superoxide as it dismutates the superoxide anion to hydrogen peroxide and oxygen. Catalase protects SOD by converting hydrogen peroxide to water and oxygen (Ighodaro and Akinloye, 2017). In this study, aluminum chloride exposure significantly decreased the level of SOD, while treatment with levocarnitine reversed this action. Another mechanism of spatial working memory performance impairment by aluminum may be due to its interference with downstream effector molecules such as cyclic GMP involved in long-term potentiation (Canales et al., 2001).

Astaxanthin has been reported to improve spatial working memory in RAM test in *Swiss albino* mice (Al-Amin et al., 2015b, 2016, 2018b). Therefore, we compared the impact of LC with AST on aluminum-induced oxidative stress. Our findings showed that LC had a stronger effect than AST (Table 1) on most of the oxidative stress parameters. MDA and Nitric oxide levels were significantly downregulated with the combined treatment of AlCl3+LC than AlCl3+AST. This could be attributed to the role of levocarnitine in transfer of LCFA to the mitochondria resulting in a reduced level of MDA. However, we do not know why LC has shown a greater impact on reducing the level of nitric oxide. Future study will be required to reveal the mechanism of LC on nitric oxide level in brain.

Besides hippocampus, the performance of radial arm maze task also depends on a specific circuit that connects hippocampus with prefrontal and ventral striatal cortices (Floresco et al., 1997). Therefore, we measured the level of oxidative stress parameters in the prefrontal cortex, striatum, and hippocampus. Moreover, we included the hypothalamus, parietal cortex and cerebellum tissues since a previous study included these brain regions and showed that levocarnitine treatment showed the neuroprotective effect by reducing the lipid peroxidation (Rani and Panneerselvam, 2002). We acknowledge that we have some experimental limitations as we could not perform immunohistochemistry and measure signaling proteins, such as NOS, HO-1 and Nrf-2 in the brain tissues, which could add value to this study. Nevertheless, we have confirmed some previous findings of the neurotoxic effect of aluminum and here we first time demonstrate the compelling evidence in supporting the beneficial effect of levocarnitine in the improvement of the brain oxidative stress and memory function in aluminum chloride-treated mice.

## Conclusion

This study aimed to investigate the effect of levocarnitine on brain oxidative stress induced by aluminum chloride. We focused on six specific brain regions namely frontal cortex, striatum, parietal cortex, hypothalamus, hippocampus, cerebellum that are distinguishable and responsible for vital brain functions. Four non-enzymatic and two enzymatic antioxidant markers were evaluated. The results obtained in this study demonstrate that treatment with levocarnitine alleviates oxidative stress as determined by evaluating oxidative stress markers in most of the brain regions and improves aluminum chloride-induced memory dysfunction.

## Data Availability

All datasets generated for this study are included in the manuscript and/or the supplementary files.

## Author Contributions

MA-A, MC, and AS performed behavioral experiments and carried out neuronal oxidative stress study. The manuscript was drafted by MA-A and MC. MA-A, AS, and MNA analyzed the data. HR, PJ, MH, MAA, and MA-A contributed to editing and critically revising the manuscript for important intellectual content. MK, AA, MR, AAh, and HR supported with chemical/lab facilities corrected drafts and obtained the funding. All co-authors approved the final version of the manuscript for submission.

## Conflict of Interest Statement

The authors declare that the research was conducted in the absence of any commercial or financial relationships that could be construed as a potential conflict of interest.
